# The Future of Measuring Impact against Malaria: From Saving Lives to Eliminating Transmission

**DOI:** 10.4269/ajtmh.17-0520

**Published:** 2017-09-27

**Authors:** Richard W. Steketee, Bernard L. Nahlen

**Affiliations:** 1 Malaria Control and Elimination Partnership in Africa (MACEPA), PATH, Seattle, Washington;; 2 President’s Malaria Initiative (PMI), US Agency for International Development (USAID), Washington, District of Columbia

At the 1998 outset of the Roll Back Malaria (RBM) Partnership, malaria was a known major cause of deaths globally, particularly among children in sub-Saharan Africa. RBM was built on the premise that the available package of proven malaria interventions, if taken to high coverage in countries, would lead to marked reductions in child mortality in Africa and overall improvement in health and well-being. The premise also highlighted the need to document progress and impact to demonstrate to external donors and national governments that financing malaria burden reduction would provide a great return on investment. The impact evaluation methods described in these reports evolved from a consensus process that engaged key RBM partners: endemic countries in Africa; the World Health Organization (WHO); The United Nations Children’s Fund (UNICEF); the President’s Malaria Initiative (PMI); the Global Fund to Fight AIDS, Tuberculosis, and Malaria (GF); and partners involved in the implementation and measurement of inputs, outcomes, and impact.^1^ This collaboration was a crucial step in directing efforts to improve systems for data collection and use. The continued ability to track progress on malaria intervention scale-up and report on impact will remain central to sustaining and increasing the funding required to meet the Sustainable Development Goal targets for malaria and move us closer to a malaria-free world.

The reports presented here provide further evidence of the impact that has occurred in many African countries—supported in particular by the countries, PMI, the GF, and many other donors and implementing partners. This remarkable success story highlights the benefit for those most at risk, African children. It convincingly shows that the core malaria interventions, when deployed at increasing coverage rates, save lives—and not just a few; there has been an estimated 60% overall reduction of deaths since 2000 in children under 5 years of age.^2^ Many children are alive today because of this work.

The initial RBM focus was on Africa, where transmission was the most intense and where the vast majority of malaria infections and deaths occurred. The core measure was all-cause child mortality (ACCM), the metric already used and regularly measured in essentially all countries by the broader child survival community. Alternative measures of “malaria-specific” or “malaria-associated”† deaths were considered; however, the diverse definitions, challenging diagnostic requirements, and lack of standard collection methods made them far from ideal for tracking impact. In Africa, there was ample evidence that malaria-specific and malaria-associated deaths were major contributors to ACCM, so the impact of malaria interventions on ACCM would be highly visible and more readily measureable. The agreed approach to monitoring changes in malaria-associated mortality using ACCM requires a plausibility argument^3^ (i.e., an assumption that mortality reductions can be attributed to programmatic efforts if improvements are found in steps along the causal pathway between intervention scale-up and mortality trends, while accounting for other contextual factors known to influence child survival).

ACCM was not the only measure needed. Data were required on the coverage of malaria interventions and the changes in other measures that were either malaria specific (e.g., parasite prevalence or case rates) or malaria associated (e.g., anemia in young children). Data to track changes in other diseases, risks, or intervention coverage were needed for the many other contributors to child death. Quality methods and data from population-representative surveys were available (e.g., demographic and health surveys^4^ and UNICEF multiple indicator cluster surveys^5^) and were supplemented through the development of the RBM malaria indicator survey^6^ that could be deployed at alternate intervals and at the peak of malaria transmission season. More recently, national health information systems have improved in quality and timeliness in some countries and, if quality can be maintained, these systems could permit reliable tracking of malaria case data. As noted in the manuscripts, additional data—including climate and geopositioning—have added value to the impact assessments, improving the robustness of the evaluations.

The success of a decade and a half of this major collaborative effort is summarized in recent UNICEF, WHO, and academic reports.^7–12^ However, the country reports in this supplement along with other recent reports using similar methods^13–15^ provide a more thorough evaluation of saving child lives amidst the broader efforts of improved immunization coverage, nutrition and micronutrient supplementation, diarrheal and respiratory disease control, education, socioeconomic status, and other factors.

The work presented here focuses on the decade from 2000 to 2010. By 2015, as per WHO^16^ and the United Nations Inter-agency Group for Child Mortality Estimation,^17,18^ the under-five mortality rate in low-income countries had decreased by 53% overall, but remains approximately 11 times the average rate found in high-income countries (seven deaths per 1,000 live births). The population coverage for vector control, effective case management, and prevention in pregnancy certainly increased in the first decade of RBM (2000 and 2010)^8,19^ and has continued to increase between 2010 and 2015.^20,21^ However, the most recent estimates for Africa show an unacceptably high number of malaria deaths associated with the persistent coverage gap: 43% of people at risk of malaria were not protected by either insecticide-treated nets or indoor residual spraying,^20^ 69% of women did not receive preventive treatment during their most recent pregnancy,^10^ and as many as 80% of children with recent fever and *Plasmodium falciparum* infection did not receive any artemisinin combination therapy.^10,21^ The progress in Africa has been achieved despite imperfect coverage; and as Dr. Tachi Yamada (then Director of Global Health at the Bill&Melinda Gates Foundation) described in 2007 when the challenge of malaria elimination and eradication was raised again, “imperfect interventions applied imperfectly can still achieve remarkable impact.”^22^

Looking forward for the next 10–15 years, continuing to close the coverage gap in high burden countries will be the key to overall success. Although we see progress in many places, 13 countries account for 76% of malaria cases and 75% of malaria deaths globally.^2^ There are a variety of reasons for their continued burden and lack of progress—insufficient resources, weak health infrastructure, inattentive leadership, social and political unrest, and other factors. For these countries and others with remaining moderate to high child mortality rates, the continued focus on the current impact evaluation methods (using ACCM and focusing on improved malaria intervention coverage in the context of improvements through other disease control programs) may be fully appropriate.

Although progress has not been uniform, some countries have done very well with intervention coverage and saving lives. Eleven high-mortality African nations reduced their under-five mortality rates by more than 50% during the 20-year interval from 1990.^23^ Countries with demonstrated success will need continued support to maintain and grow this progress. In these areas that attained very low malaria-associated mortality rates, it will no longer be sufficient to track gradual improvement in intervention coverage with the anticipation of more lives saved. The principal measure of their next stage of impact will be achieving low clinical case rates and low infection rates, perhaps en route to elimination. Transitioning to new and relevant metrics for their evolving progress remains critically important.

If we are up to the challenge of achieving, measuring, and telling the story of continued improvement toward elimination, our future will be one of continued changes.

First, the audience has broadened. At the outset of RBM, major global donors were the primary audience. The donors responded and should take great pride in the accomplishments achieved and should sustain their support to further accelerate progress. The most successful countries benefitted from that global generosity and now are being asked to contribute their own resources to address the problems that remain. At the national level, individuals and communities affected by malaria must make it known that they value the progress and want their governments to continue to push toward malaria-free regions. The private sector has also realized gains though expanded malaria control, and in some instances, companies are funding intervention scale-up for their employees. Therefore, the message must additionally engage country and business leaders and communities, and the measures of further progress must be palpable and visualized locally for this change to take hold.

Second, measurement and communication technologies have and will continue to improve dramatically. With the rollout of more effective diagnostic tests, we are now counting “confirmed cases” and not simply “fever cases” or “suspect cases.” Many countries have invested in community outreach that includes testing for malaria and treating confirmed cases. When aligned with improved reporting to the health information system, this outreach is finding and reporting a higher proportion of the overall confirmed cases. More countries have begun to roll out a common open source software platform for their health information system to assemble, analyze, and disseminate data—District Health Information Software^24^—and improvements in communication technology permit moving data into that system much more rapidly. As countries scale up their intervention coverage, decrease transmission, and count fewer cases, they will be increasingly able to respond to the individual cases with investigation and local transmission containment. The further strengthening of health information systems to collect credible and timely data, analyze and visualize the information, and take action will be an absolute requirement for elimination and is described as one of the pillars of the Global Technical Strategy.^25^ Thus, programs must start early to build the information system that can accomplish these tasks and inform the use of all antimalarial tools.

Third, major efforts to develop the next generation of tools are beginning to bear fruit. With better diagnostics, effective antimalarial drugs, improved insecticide-based or novel vector control methods, vaccines, and improvements in the information systems, each will need to find their place in the intervention package. Given the new tools, the expanding audiences, and the progress already achieved in saving lives, a central question remains: “what compelling measures and targets can we use to celebrate the progress that will occur in that program interval between saving lives and eliminating malaria?” Experience with the transition from malaria control to malaria elimination suggests that the malaria deaths go away relatively early and the infections linger, so there will be a long time in the end game as countries strive to measure “zero.”

Finally, although many aspects will change, the critical need for high-quality, timely, informative, and actionable data remains. The early collaboration among RBM partners developed consensus on a common approach to impact evaluation, standardization of household survey methodology, and common data elements for collection through routine health information systems; these continue to make in-country, cross-country, and global consensus measurement possible. The reports here are based on this consensus approach for impact evaluation and sum to a strong body of compelling information. As the authors of these reports note, much has been learned and refined in the process.^26^ Given the changing audience, tools, measurement, communications, and the evolving differences between countries, commitment and support for this consensus process and for the collection and use of quality and timely data will be ever more critical for sustained credible progress against malaria.

## Footnotes

† “Malaria-associated” deaths may have another primary or concurrent cause, but malaria likely contributed substantially to the risk of death: for example, severe malaria-associated anemia contributing to death in a child with lower respiratory infection.
